# Diabetes Mellitus with Influenza Virus and Subsequent Bacterial Infection: Triple Pathological Interactions and Challenges

**DOI:** 10.3390/ijms27156539

**Published:** 2026-07-23

**Authors:** Huiqiang Wang, Yuhuan Li

**Affiliations:** 1 Beijing Key Laboratory of Technology and Application for Anti-Infective New Drugs Research and Development, Institute of Medicinal Biotechnology, Chinese Academy of Medical Sciences and Peking Union Medical College, Beijing 100050, China; 2 NHC Key Laboratory of Biotechnology of Antibiotics, Institute of Medicinal Biotechnology, Chinese Academy of Medical Sciences and Peking Union Medical College, Beijing 100050, China; 3 Division for Medicinal Microorganism-Related Strains, CAMS Collection Center of Pathogenic Microorganisms, Beijing 100050, China

**Keywords:** diabetes mellitus, influenza virus, subsequent bacterial infection, pathogenesis, clinical management

## Abstract

Diabetes mellitus (DM), characterized by persistent hyperglycemia, not only induces chronic damage and dysfunction across multiple tissues and organs but also increases susceptibility to a broad spectrum of pathogens. Diabetes serves as a significant risk factor for influenza virus infection and associated secondary bacterial pneumonia. This review mainly focuses on the complex pathological and physiological interactions among type 2 diabetes mellitus, influenza and bacterial infections. Chronic hyperglycemia and the resulting immune–metabolic disorders lead to impaired innate and adaptive immune functions. Influenza virus infection further compromises the respiratory barrier, induces an excessive inflammatory response, and depletes immune cells, thereby creating an ideal microenvironment for bacterial colonization. The ensuing bacterial infection synergizes with the virus, forming a vicious cycle of pathogenesis. An integrated strategy combining optimized glycemic control, enhanced vaccination, and early, precise antiviral and antibacterial therapy is paramount for the prevention and treatment of secondary bacterial infections following influenza in patients with diabetes mellitus.

## 1. Introduction

**Figure 1:**
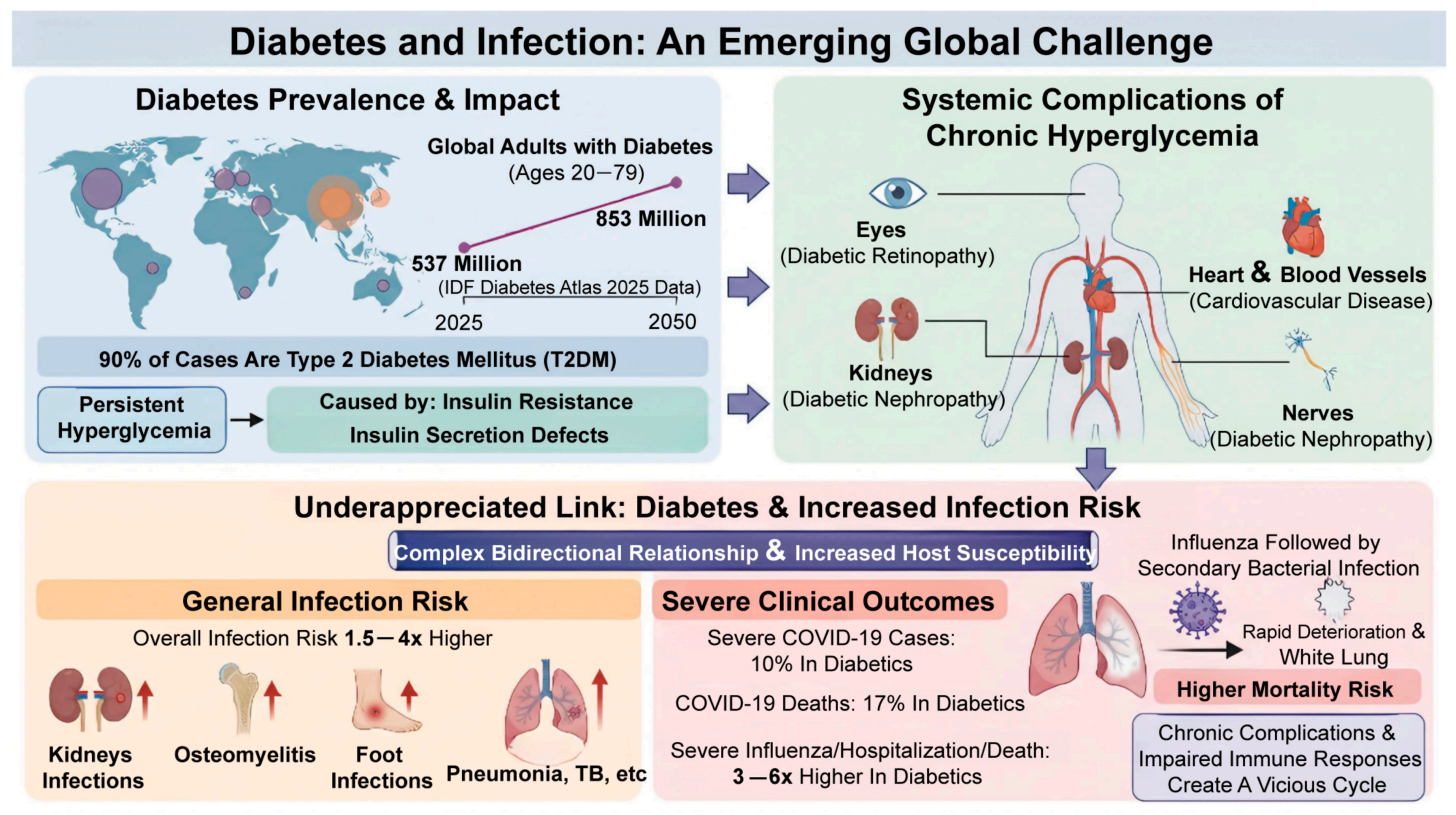
Diabetes and infection: an emerging global challenge. Diabetes mellitus, primarily type 2 (>90%), is a major global health challenge, affecting 537 million adults. Characterized by insulin resistance and/or secretion defects, chronic hyperglycemia drives multi-organ damage. Crucially, diabetes increases susceptibility to pathogens (1.5- to 4-fold) and worsens infection outcomes. The figure was created using PicDoc Web version and Adobe Illustrator 2023 software [1,2,3,4,5,6,7,8,9,10,11,12,13,14,15,16,17].

Diabetes has become one of the most serious global public health challenges in the 21st century. The latest International Diabetes Federation (IDF) Diabetes Atlas (2025) reports that there are currently 537 million people aged 20 to 79 suffering from diabetes worldwide (Figure 1). It is predicted that this number will reach 853 million by 2050, with type 2 diabetes mellitus (T2DM) accounting for over 90% [1]. T2DM is characterized by persistent hyperglycemia resulting from insulin resistance, defects in insulin secretion, or a combination of both. Chronic hyperglycemia leads to long-term damage, dysfunction, and failure of various organs and tissues, primarily affecting the eyes (diabetic retinopathy), kidneys (diabetic nephropathy), heart and blood vessels (cardiovascular disease), and nerves (diabetic neuropathy) (Figure 1) [2]. It is a leading cause of blindness, renal failure, myocardial infarction, stroke, and lower limb amputation [2].

However, a persistently underestimated yet increasingly prominent issue is the complex and intimate relationship between T2DM and infectious diseases. T2DM significantly increases susceptibility to a wide range of pathogens. Epidemiological data indicate that individuals with diabetes have a 1.5- to 4-fold higher risk of both common and severe infections (Figure 1) [3,4,5,6]. This elevated risk is most pronounced in renal infections, osteomyelitis, and foot infections, while the risks of pneumonia, tuberculosis, and skin and soft tissue infections are also markedly increased (Figure 1) [3,4,5]. Of particular concern is the markedly worsened clinical outcome once infection occurs in individuals with T2DM. As exemplified by the COVID-19 pandemic, diabetes mellitus has been estimated to account for 10% of severe COVID-19 cases and 17% of COVID-19-related deaths globally (Figure 1) [7].

Influenza is a highly contagious acute respiratory disease that breaks out globally each year and also causes periodic pandemics. According to the World Health Organization, there are approximately one billion cases of seasonal influenza annually, including 3 to 5 million severe cases [8]. Seasonal influenza epidemics lead to an estimated 290,000 to 650,000 deaths worldwide each year [9]. Older adults, children, chronic disease population, and pregnant women are more susceptible to progressing to severe illness following influenza infection, with a significantly higher risk of influenza-associated mortality compared to the healthy population [10]. Although most infections caused by influenza A viruses (IAV) are not fatal, secondary bacterial infections represent a critical factor contributing to worse clinical outcomes and increased hospitalization rates [11,12,13]. Bacterial infections typically occur within several days following influenza infection. Compared to viral infection alone, such co-infections can lead to rapid clinical deterioration within a short period, potentially resulting in conditions such as “white lung” (severe pulmonary infiltration) and respiratory failure, thereby significantly elevating the risk of mortality [11,14,15]. Epidemiological data clearly indicate that individuals with T2DM face a 3 to 6 times higher risk of progressing to severe illness, requiring hospitalization, and experiencing mortality following influenza infection compared to the non-diabetic population [16,17]. The multiple defects in immune defense among individuals with diabetes not only predispose them to higher susceptibility to influenza infection but also impair their ability to clear the virus, ultimately rendering them “ideal hosts” for secondary bacterial infections. Therefore, deepening the understanding of the triple interactions among diabetes mellitus, influenza virus, and bacteria is of great significance for reducing the morbidity and mortality of related infections in patients with diabetes.

This review aims to unravel current research progress on the intricate relationships among diabetes, influenza virus infection, and secondary bacterial infection. It will provide an in-depth analysis of how diabetes systematically reshapes the host immune status, creating a susceptible “soil” for infections. This review summarizes current understanding of the molecular mechanisms underlying respiratory barrier disruption and immunopathological damage following influenza virus infection, and critically discusses the proposed biological basis for viral–bacterial synergistic pathogenicity based on available experimental evidence. Finally, recommendations are proposed for the prevention and treatment of influenza complicated by bacterial infections in patients with T2DM.

## 2. Hyperglycemia as the Underlying Condition for Infection Susceptibility

### 2.1. Hyperglycemia Compromises Mucosal Barrier Integrity

**Figure 2:**
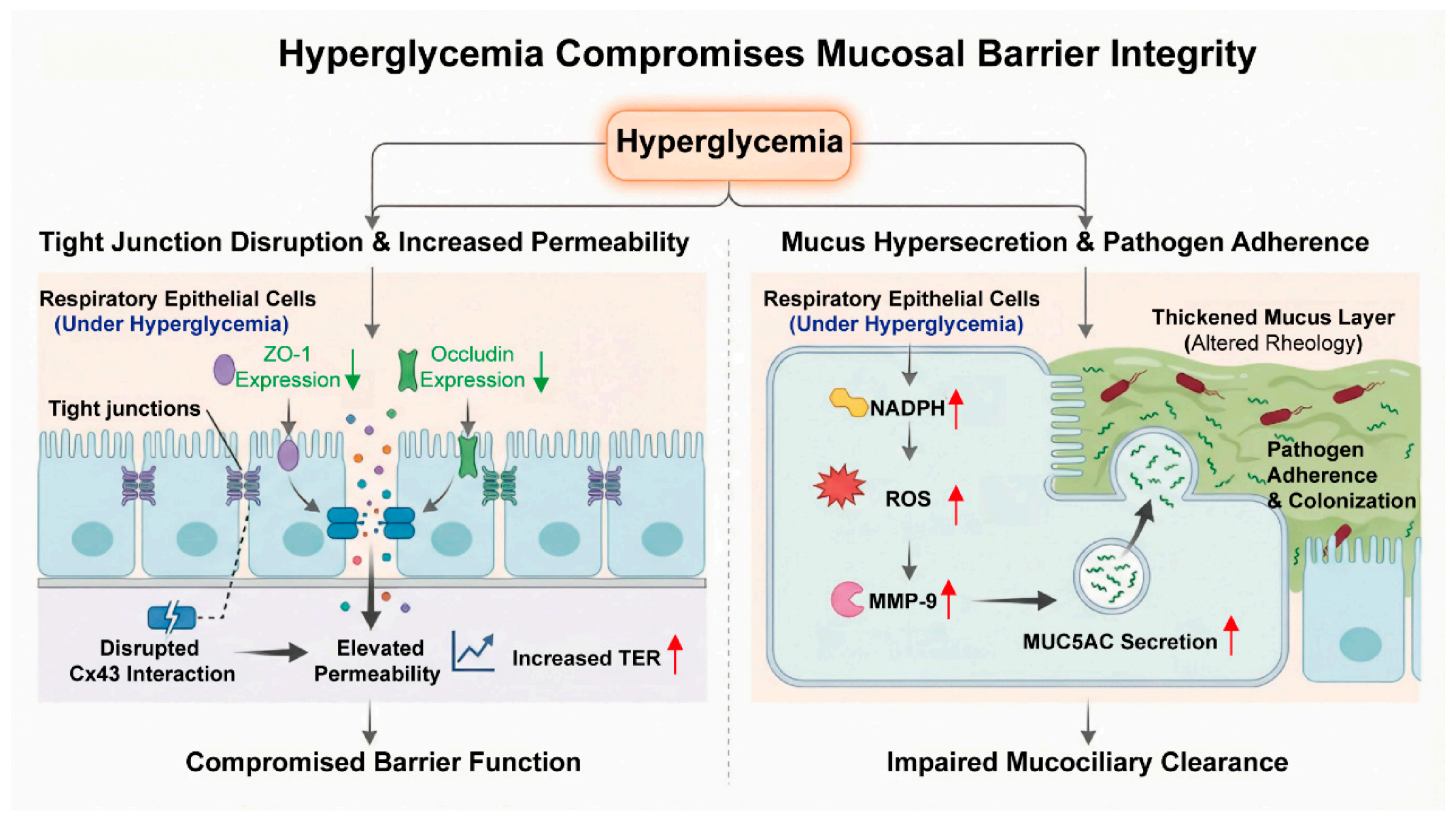
Hyperglycemia compromises mucosal barrier integrity. Under hyperglycemic conditions, the expression of tight junction proteins (e.g., ZO-1 and occludin) in respiratory epithelial cells is downregulated, which disrupts the interaction between Cx43 and tight junctions, leading to increased TER and elevated permeability. Meanwhile, hyperglycemia activates the NADPH/ROS/MMP-9 signaling pathway, inducing excessive secretion of the mucin protein MUC5AC. The altered mucin rheological properties result in thickened mucus that is difficult to clear, thereby promoting pathogen adherence and colonization. The figure was created using PicDoc Web version and Adobe Illustrator 2023 software [18,19,20,21].

To defend against microbial invasion, the lungs have developed a sophisticated physical defense mechanism. The airway epithelial physical barrier comprises both the production of mucus on mucosal surfaces and a robust mucociliary blanket dedicated to the clearance of microorganisms [18]. Hyperglycemia can lead to decreased expression of tight junction proteins (such as ZO-1 and occludin) in respiratory epithelial cells. This disrupts the interaction between Connexin 43 (Cx43) and tight junctions, thereby increasing transepithelial electrical resistance (TER) and permeability in human airway epithelial cells (Figure 2) [19]. The hyperglycemic environment induces excessive production of the mucin protein MUC5AC in human respiratory epithelial cells through activation of the nicotinamide adenine dinucleotide phosphate (NADPH)/reactive oxygen species (ROS)/Matrix Metalloproteinase-9 (MMP-9) signaling pathway [20,21]. This mucus hypersecretion alters mucin rheological properties, rendering it thick and difficult to clear effectively, thereby facilitating pathogen adherence and colonization (Figure 2) [20,21]. Influenza and subsequent bacterial infection can further exacerbate the damage to the tight junction protein ZO-1, thereby more severely compromising the integrity of the pulmonary epithelial barrier. This facilitates easier pathogen invasion and deeper dissemination into lung tissues, ultimately aggravating the infection. While the detrimental effects of hyperglycemia on epithelial barrier integrity are well documented in in vitro and animal models, it should be noted that most studies have employed supraphysiological glucose concentrations that may not fully recapitulate the dynamic glycemic fluctuations seen in clinical settings. It is currently unclear whether strict blood sugar control can reverse these barrier defects in humans. Further prospective clinical studies are needed to confirm this.

### 2.2. Hyperglycemia Impairs Innate Immunity

The first line of immunological defense against invading pathogens is the innate immune system, which comprises various immune cells such as macrophages, neutrophils, and natural killer (NK) cells. Chronically elevated blood glucose levels directly impair the functions of multiple immune cell types. Hyperglycemia-induced insulin resistance not only affects glucose utilization but also disrupts the energy metabolism of immune cells. Upon activation, immune cells such as macrophages and T cells undergo a dramatic metabolic shift from oxidative phosphorylation to glycolysis (known as the “Warburg effect”) to meet the high energy demands for rapid proliferation and effector functions [22,23]. Hyperglycemia-induced insulin resistance undermines this metabolic reprogramming through several mechanistically distinct pathways. First, impaired insulin receptor substrate (IRS) phosphorylation and subsequent PI3K/AKT signaling activation, even in the presence of hyperglycemia, directly attenuate mTOR complex 1 (mTORC1) activity and impair the glycolytic induction necessary for immune cell activation [24]. Second, insulin resistance is associated with mitochondrial dysfunction, characterized by impaired mitochondrial biogenesis, compromised oxidative phosphorylation, and excessive generation of mitochondrial reactive oxygen species (mtROS), which further exacerbates oxidative damage and hampers the metabolic plasticity required for appropriate immune responses [25].

**Figure 3:**
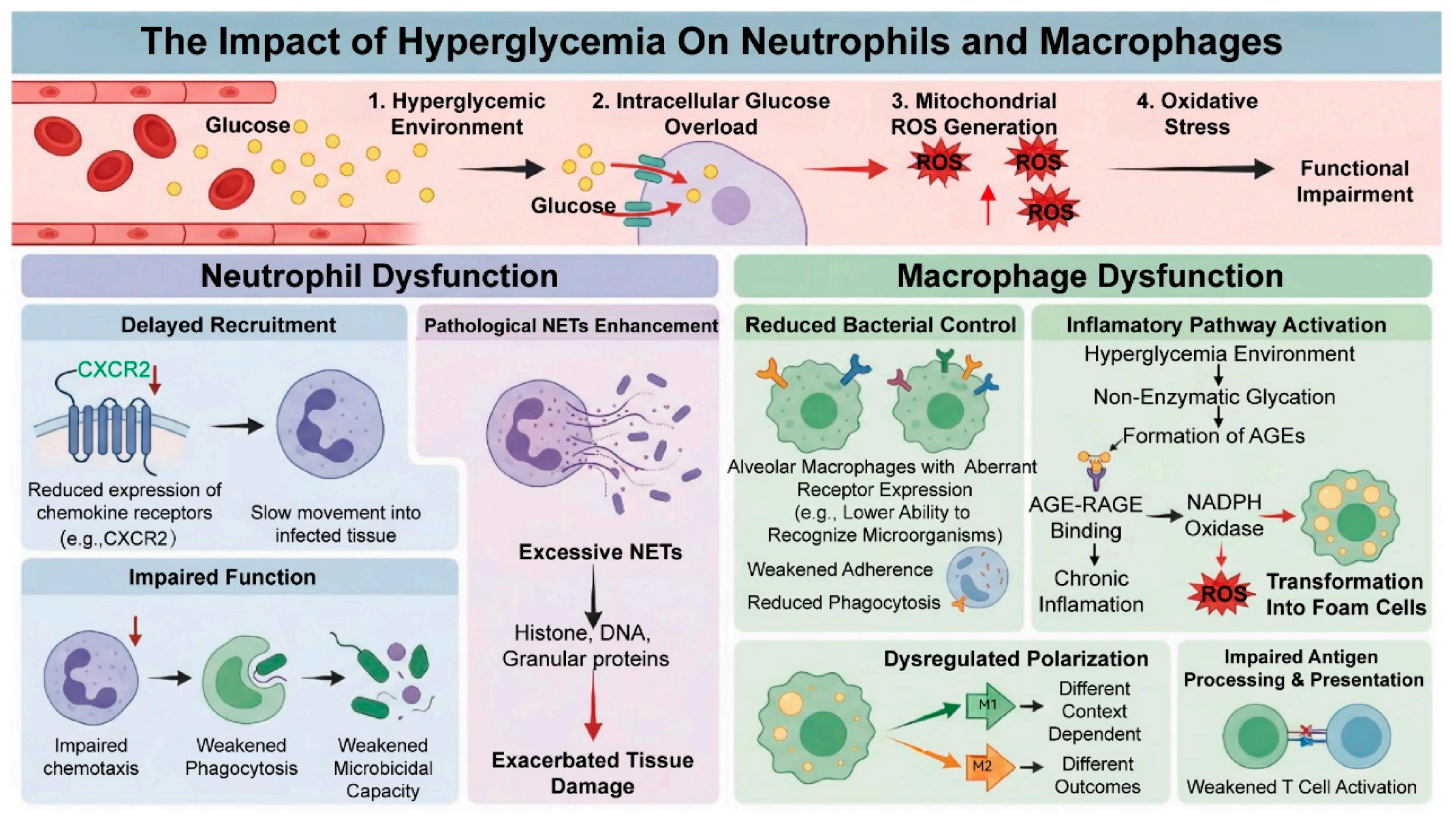
The impact of hyperglycemia on the functions of neutrophils and macrophages. Under hyperglycemic conditions, the increased formation of ROS and AGEs leads to oxidative stress, which damages the functions of cells such as chemotaxis, phagocytosis, etc. Furthermore, hyperglycemia can lead macrophages into a chronic, low-grade inflammatory state, causing polarization disorders in macrophages and promoting their transformation into macrophage foam cells. The figure was created using PicDoc Web version and Adobe Illustrator 2023 software [22,23,24,25,26,27,28,29,30,31,32,33,34,35,36,37].

In neutrophils, reactive oxygen species (ROS) are generated as a key defense mechanism, serving dual roles: they act as direct microbicidal effectors within phagolysosomes during phagocytosis, and function as signaling molecules that initiate the formation of neutrophil extracellular traps (NETs) [26]. A hyperglycemic environment leads to intracellular glucose overload, which drives mitochondria to generate excessive ROS. This triggers oxidative stress and impairs the chemotactic, phagocytic, and microbicidal functions of neutrophils (Figure 3) [26,27]. Impaired neutrophil function manifests as reduced expression of chemokine receptors (such as CXCR2), resulting in delayed and diminished recruitment to infection sites, as well as weakened phagocytic capacity (Figure 3) [28]. Paradoxically, this dysfunction is accompanied by a pathological enhancement in NETs formation, which in turn exacerbates tissue damage (Figure 3) [29,30,31].

A hyperglycemic environment can cause aberrant expression of receptors and adhesion molecules on the surface of alveolar macrophages, thereby reducing their ability to recognize and adhere to pathogenic microorganisms [32]. Furthermore, a hyperglycemic environment can significantly increase the level of ROS in macrophages, thereby triggering oxidative stress (Figure 3) [33]. Hyperglycemia promotes non-enzymatic glycation of proteins, leading to the formation of advanced glycation end-products (AGEs) (Figure 2). These AGEs further induce nicotinamide adenine dinucleotide phosphate (NADPH) oxidase to generate ROS, which impair macrophage function and facilitate their transformation into macrophage foam cells [34,35]. The binding of AGEs to their receptor RAGE leads to sustained activation of inflammatory pathways such as nuclear factor-kappa B (NF-κB), resulting in a chronic low-grade inflammatory state (Figure 3) [36,37]. Hyperglycemia leads to dysregulated macrophage polarization, and its polarization direction is highly dependent on the specific disease context and tissue microenvironment [38,39]. Additionally, the ability of macrophages to process antigens after phagocytosis and to initiate adaptive immune responses is also impaired [40]. In summary, chronically elevated blood glucose levels impair the functions of multiple immune cell types, thereby compromising innate immunity.

### 2.3. Hyperglycemia Impairs Adaptive Immunity

The adaptive immune response is primarily initiated by dendritic cells (DCs) residing in the airways and interstitium of the lungs. These cells drive the proliferation and differentiation of virus-specific T cells and B cells [41,42]. Impairment of adaptive immunity due to hyperglycemia is also significant. As key antigen-presenting cells, DCs exhibit suppressed maturation and migratory capacity under high-glucose conditions, leading to inadequate activation of T cells. Studies have shown that hyperglycemia impairs the expression of co-stimulatory molecules, antigen transport, and T-cell priming across different pulmonary DCs subsets [43]. These dysfunctions contribute to deficient antiviral adaptive immune responses, delayed viral clearance, and increased mortality. Hyperglycemia induces mitochondrial dysfunction and aberrantly increased fatty acid synthesis in CD4 (+) T cells, resulting in elevated cellular oxidative stress and abnormal lipid accumulation [44]. This promotes lipid peroxidation, impairs the differentiation of CD4 (+) T cells into T helper 1 (Th1) cells, directly leads to inadequate T-cell activation, weakens cellular immunity against intracellular pathogens and mucosal defense, and ultimately results in failure to initiate an adaptive immune response [44]. In patients with T2DM, CD8 (+) T cells, particularly the highly differentiated CD8 (+) EMRA subset, exhibit mitochondrial dysfunction, including impaired energy production, increased mitochondrial reactive oxygen species (mtROS), compromised fatty acid oxidation, and fragmented mitochondrial morphology [45]. These abnormalities significantly diminish their ability to clear virus-infected cells. Under hyperglycemic conditions, the normal glucose metabolism of B cells is disrupted, such as GLUT1-mediated uptake [46]. This impairment compromises the germinal center response, leading to defective antibody affinity maturation and inadequate plasma cell generation [44]. Consequently, it results in reduced affinity, diminished production, and shortened persistence of antibodies against pathogens or vaccines. In T2DM, this manifests as a generalized impairment in the response to foreign antigens, whereas in T1DM, B-cell dysfunction is primarily characterized by aberrant activation toward self-antigens and pathogenic antibody class switching [47]. In summary, impairment of adaptive immunity leads to lower response rates and faster waning of protective efficacy to vaccines in individuals with diabetes.

Collectively, the evidence indicates that hyperglycemia broadly impairs both innate and adaptive immunity, but notable knowledge gaps persist. First, most mechanistic studies have been conducted in murine models or in vitro systems, and the translational relevance to human diabetic patients requires further validation. Second, the relative contribution of innate versus adaptive immune defects to infection susceptibility remains poorly quantified. Addressing these gaps will be essential for developing precision prevention strategies for diabetic patients.

### 2.4. Hyperglycemia-Induced Inflammaging Results in Impaired Immune Priming and Dysregulated Immune Responses

The chronic low-grade inflammatory state induced by hyperglycemia, known as “inflammaging,” often manifests as a paradoxical phenomenon during acute infections: an initial sluggish response followed by later uncontrolled inflammation. The sluggish response occurs because chronic low-grade inflammation depletes immune resource reserves, leading to a weakened and delayed early antiviral response upon encountering a new infection, such as reduced production of type I interferons. Prolonged exposure to inflammatory cytokines impairs the function of tissue-resident immune cells, including alveolar macrophages, diminishing their ability to recognize and clear pathogens. Furthermore, sustained chronic stimulation desensitizes pattern recognition receptor signaling pathways, such as those mediated by Toll-like receptors, raising the threshold for responding to acute infections [48]. Under the influence of chronic inflammation, hematopoietic stem cells exhibit a skewed differentiation toward the myeloid lineage, and the newly generated immune cells themselves also harbor functional impairments [49,50]. Uncontrolled inflammation is not merely an extension of chronic inflammation but a distinct pathological state. In chronic inflammation, a persistent low-grade pro-inflammatory milieu is established. In uncontrolled inflammation, however, specific pro-inflammatory pathways (such as NF-κB) exhibit elevated baseline activity, while negative regulatory mechanisms are impaired, including IL-10/TGF-β feedback loops and regulatory T-cell function. This net pro-inflammatory bias predisposes to an excessive and poorly restrained response upon acute infectious challenge [51]. Once an acute infection occurs, it readily triggers an excessive and dysregulated inflammatory response, leading to tissue damage and cytokine storm.

## 3. The Initial Impact of Influenza Virus Infection

Influenza virus infection itself constitutes a profound immunostress process. The virus binds to sialic acid receptors on the surface of respiratory epithelial cells via hemagglutinin (HA), enters the cells for replication, and causes direct cellular damage and apoptosis [52,53]. Virus-associated molecular patterns released from infected cells are recognized by host cells, triggering the production of type I interferons and a large number of pro-inflammatory cytokines [54,55]. In diabetic patients, this process is significantly amplified. Studies have shown that diabetic mouse models infected with the influenza virus exhibit higher pulmonary viral loads and delayed viral clearance [43,56]. This is attributed to the aforementioned delay in innate immune recognition and response. More importantly, the pre-existing chronic inflammatory state and metabolic disturbances in diabetic hosts make the inflammatory response to influenza virus highly prone to loss of control. This manifests as an excessive cytokine storm, causing widespread damage to alveolar epithelium and vascular endothelium, leading to alveolar edema, hemorrhage, and hyaline membrane formation, constituting the pathological basis of severe viral pneumonia [57]. Such intense tissue destruction not only severely impairs gas exchange function but also fundamentally undermines the first line of physical and immune defense of the respiratory tract against bacterial invasion, thereby paving the way for secondary bacterial infections.

Although the amplified inflammatory response in diabetic hosts is widely accepted, the precise molecular pathways linking hyperglycemia to exaggerated cytokine production during influenza infection remain incompletely defined. In particular, the relative roles of AGE–RAGE signaling, metabolic reprogramming of immune cells, and epigenetic modifications in driving this hyperinflammatory phenotype are areas of active investigation. Furthermore, whether the heightened susceptibility of diabetic patients to severe influenza is primarily attributable to impaired viral clearance, exaggerated immunopathology, or a combination of both remains an unresolved question with direct therapeutic implications.

## 4. The Core Mechanisms Underlying Secondary Bacterial Infections

### 4.1. Virus-Induced Immunosuppression and Microenvironmental Alterations

Following influenza virus infection, the host immune system enters a transient state of immune paralysis, which serves as a critical window for secondary bacterial infections. Although type I interferons (IFN-I) are central to antiviral defense, their persistently elevated levels during the later stages of infection can paradoxically impair protective antibacterial immunity [58,59].

**Figure 4:**
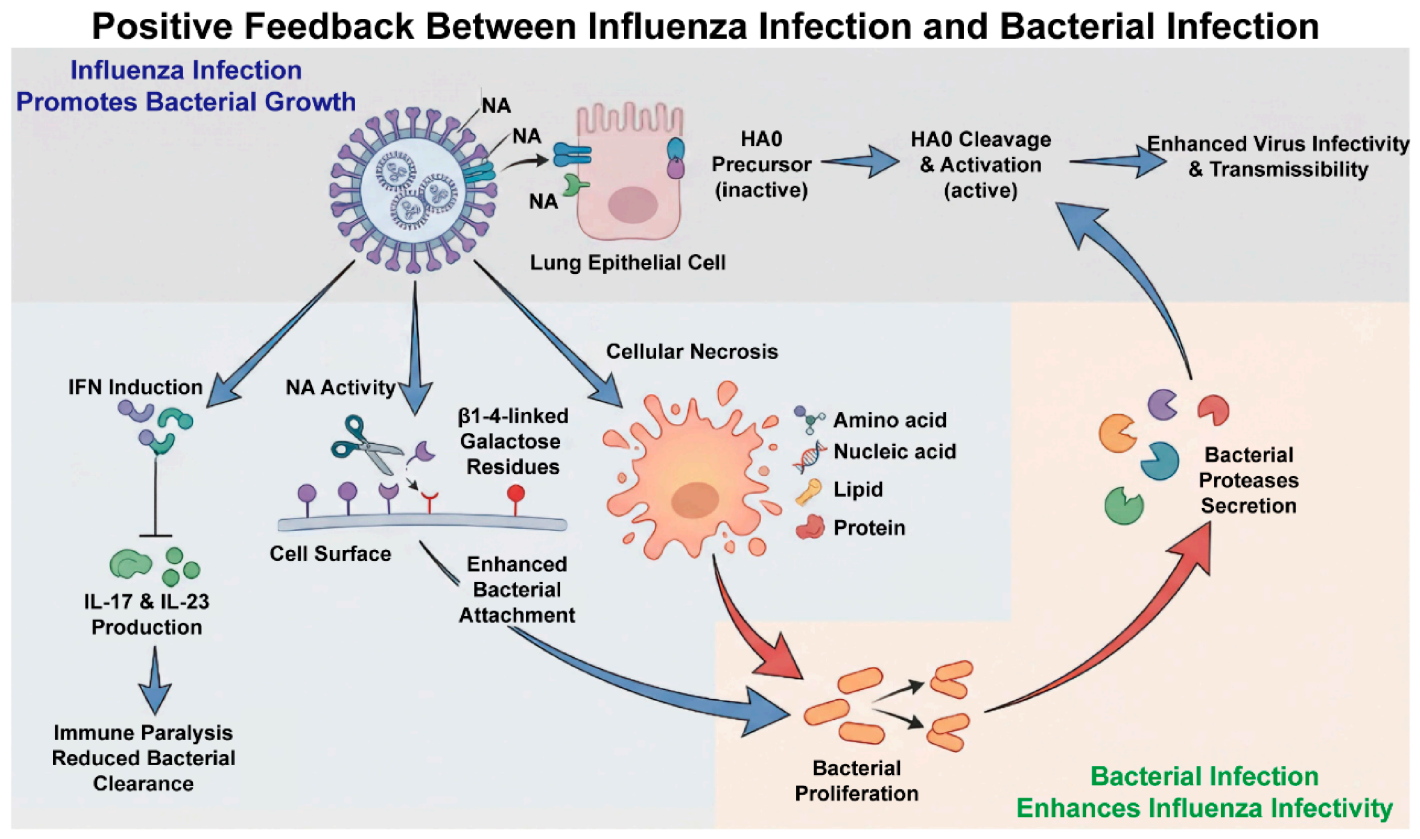
Positive feedback between influenza infection and subsequent bacterial infection. The interferon induced by influenza infection inhibits the production of IL-17 and IL-23, causing immune paralysis and preventing the lungs from initiating effective monitoring and clearing bacterial infections. The NA protein of influenza can cleave and remove the sialic acid residues on the cell surface, exposing the β1-4-linked galactose residues, thereby facilitating the attachment of bacteria. Influenza infection leads to massive necrosis of lung epithelial cells, releasing cellular debris containing amino acids, nucleic acids, lipids, and proteins, all of which provide direct carbon and nitrogen sources for bacterial proliferation. Bacteria secrete various bacterial proteases, which can efficiently cleave and activate the HA0 precursor of the influenza virus, thereby enhancing the infectivity and transmissibility of the virus and forming a positive feedback loop. The figure was created using PicDoc Web version and Adobe Illustrator 2023 software [58,59,60,61,62,63,64,65,66,67,68,69,70,71].

A key mechanism through which influenza virus undermines antibacterial host defense involves the IL-23/IL-17 axis. In the healthy lung, IL-23, primarily produced by antigen-presenting cells including alveolar macrophages and dendritic cells, promotes the differentiation and maintenance of Th17 cells, which in turn secrete IL-17 [60,61]. IL-17 subsequently stimulates epithelial cells to produce antimicrobial peptides and chemokines, facilitating the recruitment and activation of neutrophils, a critical early response for bacterial clearance [62,63,64]. Influenza virus infection disrupts this protective axis through multiple interconnected mechanisms. First, excessive IFN-I signaling directly suppresses IL-23 production by antigen-presenting cells and inhibits Th17 cell differentiation [65]. Second, influenza virus-induced attenuation of IL-1β production further compromises Th17 polarization, as IL-1β is an essential co-stimulatory signal for Th17 development [66]. Third, the profound depletion of alveolar macrophages, a major source of pulmonary IL-23, following viral infection diminishes the local capacity to initiate Type 17 responses [67]. Consequently, IL-17 and IL-23 levels are significantly reduced during influenza virus infection, as demonstrated in murine models of post-influenza *Staphylococcus aureus* pneumonia [65,66]. The resultant impairment of neutrophil recruitment and antimicrobial peptide production renders the lungs unable to mount effective alarm and clearance programs against invading bacteria. Thus, the IFN-I-dominated environment during the convalescent phase of influenza essentially creates an ‘immunological blind spot’ that favors secondary bacterial colonization and dissemination (Figure 4). Despite the compelling evidence for IFN-I-mediated suppression of Type 17 responses, several important controversies remain. First, the relative contribution of IFN-I versus other viral factors to IL-23/IL-17 axis suppression has not been systematically dissected. Second, while murine models demonstrate IL-17 reduction following influenza infection, human data are more limited and somewhat conflicting, with some studies reporting elevated IL-17 levels in severe influenza. This discordance may reflect species differences, timing of sample collection, or the confounding effects of pre-existing comorbidities. Third, the extent to which these mechanisms are amplified in diabetic patients, versus being qualitatively similar to non-diabetic hosts, remains largely unexplored.

Hyperglycemia fundamentally disrupts the polarization dynamics of macrophages, a process with profound implications for pulmonary host defense. In T2DM, chronic hyperglycemia creates a persistent pro-inflammatory milieu that skews macrophage polarization toward a dysfunctional M1-like phenotype, characterized by elevated production of TNF-α and IL-1β but paradoxically reduced nitric oxide production and impaired phagocytic and bactericidal activity [68,69]. Mechanistically, sustained high glucose exposure compromises macrophage glycolytic capacity and glycolytic reserve, limiting the bioenergetic support essential for effective phagocytosis and microbial killing. The functional deterioration of alveolar macrophages in diabetes is particularly relevant to influenza-associated secondary bacterial infections. Diabetic mice exhibit not only reduced alveolar macrophage numbers but also impaired mitochondrial function and compromised phagocytic capacity, which correlate directly with increased susceptibility to influenza A virus infection [68]. During influenza virus infection, the already dysfunctional alveolar macrophage pool is further depleted through virus-induced apoptosis and exhaustion. When secondary bacterial invasion occurs, the remaining macrophages, harboring both polarization bias and metabolic deficits, fail to mount an effective antibacterial response. Their impaired phagocytosis and reduced ROS production allow for bacterial proliferation, while their aberrant pro-inflammatory cytokine release exacerbates immunopathology. This maladaptive macrophage phenotype thus constitutes a critical link between diabetic metabolic dysregulation and the heightened risk of severe post-influenza bacterial pneumonia.

Influenza virus infection triggers an intense antiviral response, leading to massive apoptosis of lymphocytes and alveolar macrophages, resulting in immune cell exhaustion (Figure 4) [70,71]. Alveolar macrophages constitute the first line of defense in the alveolar cavity for bacterial clearance. The severe depletion and functional impairment caused by influenza infection not only compromise the clearance of viruses and apoptotic cells but also disrupt the pulmonary tissue repair barrier, thereby allowing invading bacteria to proliferate rapidly [67]. Following viral infection, direct damage to lung epithelial cells by the virus triggers a robust inflammatory response and activates tissue repair signaling [72,73]. In the context of diabetes, the repair process is often dysregulated. Excessive tissue remodeling and a propensity for fibrosis create an environment conducive to bacterial biofilm formation [74]. In conclusion, virus-induced immunosuppression and microenvironmental alterations provide a potent breeding ground for subsequent bacterial infections.

### 4.2. Molecular Basis of Viral–Bacterial Synergistic Pathogenesis

Viruses and bacteria do not act independently but exhibit direct molecular-level synergy. Influenza virus infection exposes bacterial adhesion receptors. The surfaces of healthy respiratory and alveolar epithelial cells are coated with a layer of glycoproteins and glycolipids rich in sialic acid. Sialic acid acts as a protective coating that not only partially blocks pathogens but also repels bacteria through its negative charge. When cells are infected with influenza virus, the neuraminidase (NA) protein cleaves and removes sialic acid residues from the cell surface, exposing the underlying glycan structures that were previously concealed (Figure 4) [75]. Many respiratory pathogens, such as Staphylococcus aureus and *Streptococcus pneumoniae*, possess adhesins on their surfaces that specifically recognize and bind to these exposed glycan structures. Through direct cytotoxicity or by inducing apoptosis and necrosis, the virus causes extensive shedding of alveolar epithelial cells and vascular endothelial cells, thereby exposing basement membrane components, such as fibronectin and laminin [76,77]. These components also serve as key adhesion targets for bacteria. Through this synergistic mechanism of the “virus unlocking the door and bacteria entering,” the adhesion efficiency and colonization density of bacteria in the respiratory tract are significantly enhanced, laying a solid foundation for subsequent breach of the epithelial barrier and the development of invasive infections [78].

Bacterial proteases enhance the pathogenicity of influenza viruses. The HA protein of influenza viruses is synthesized as an inactive precursor, HA0. It must be cleaved by host cell proteases into two subunits, HA1 and HA2, linked by a disulfide bond, for the influenza virus to acquire the ability to infect cells [79,80]. Staphylococcus aureus is capable of secreting various proteases. These bacterial proteases can efficiently cleave and activate the HA0 precursor of the influenza virus, thereby enhancing viral infectivity and transmission capacity and establishing a positive feedback loop of viral and bacterial infections (Figure 4) [81,82]. In addition to their direct proteolytic action on the HA of influenza virus, bacteria may also convert plasminogen into plasmin via bacterial plasminogen activators, which in turn indirectly cleaves HA and promotes the progression of influenza disease [83,84].

A metabolic bridge exists between influenza infection and bacterial infection. Influenza infection leads to massive necrosis of lung epithelial cells, releasing cellular debris containing amino acids, nucleic acids, lipids, and proteins, all of which provide direct carbon and nitrogen sources for bacterial proliferation (Figure 4) [85,86]. The inflammatory response induced by viral infection increases vascular permeability, causing plasma components rich in glucose, electrolytes, and other substances to extensively leak into the alveolar spaces. It can be expected that in diabetic patients, the exudate contains glucose concentrations far exceeding physiological levels, providing bacteria with a more efficient energy source. The hyperglycemic environment itself further exacerbates tissue inflammation, damages vascular endothelium, and impairs the function of immune cells such as neutrophils [22,43]. This results in an increased “culture medium” supply while the immune system’s ability to clear invading bacteria and necrotic debris is significantly diminished. In the context of diabetes, the metabolic bridge connecting primary viral infection and secondary bacterial infection is strengthened and widened, explaining why T2DM is a high-risk group for various infections.

The molecular synergy between influenza virus and bacteria is well established at the experimental level, but several clinically relevant questions remain unanswered. Notably, it is unclear whether the enhanced viral replication driven by bacterial proteases observed in vitro translates into clinically meaningful differences in disease progression in diabetic patients. Moreover, the metabolic bridge between viral and bacterial infection, while mechanistically plausible, has not been directly quantified in human diabetic lung tissue. Whether the hyperglycemic milieu in the alveolar space can be therapeutically targeted to disrupt this metabolic synergy represents an intriguing but untested hypothesis.

## 5. Strategies and Recommendations

The following recommendations are derived from a synthesis of available evidence, including high-level evidence from randomized controlled trials, observational studies and expert consensus guidelines, and mechanistic studies that provide rationales for consideration (Table 1). Building upon the critical synthesis above, we offer our own perspectives on the key knowledge gaps that remain unresolved and propose priority research directions that we believe will most effectively advance the field.

### 5.1. Preventive Strategies

Optimizing blood glucose management serves as the cornerstone for preventing all infectious complications. A 2024 retrospective study revealed that diabetic patients using metformin had a lower hospitalization rate following influenza-related medical visits compared to those not using metformin (56.8% vs. 70.1%), suggesting that effective glycemic control may confer additional protective benefits [87]. Beyond metformin, emerging evidence suggests that other glucose-lowering agents may differentially modulate infection risk. A systematic review and meta-analysis of real-world studies demonstrated that SGLT2 inhibitor use was associated with a significantly reduced risk of pneumonia (HR 0.61, 95% CI 0.57–0.66), pneumonia-related mortality (HR 0.49, 95% CI 0.35–0.67), and sepsis (HR 0.45, 95% CI 0.30–0.68) [88]. Additionally, a large cohort study of older adults with type 2 diabetes found that relaxed glycemic control (per guideline recommendations) did not increase hospitalization risk for most infections in older type 2 diabetic patients, except for skin, soft tissue, and bone infections at HbA1c 8% to <9% [89]. These findings collectively indicate that while optimized glycemic control is broadly beneficial, the relationship between specific glucose-lowering agents, glycemic targets, and infection outcomes is complex and warrants further investigation. Overall, robust long-term glycemic control significantly improves immune function, necessitating intensified monitoring and therapeutic regimen adjustments during influenza season.

Active vaccination is strongly recommended. Annual administration of the seasonal influenza vaccine constitutes a core preventive measure. However, standard-dose vaccines often fail to achieve adequate protection in diabetic patients with poor glycemic control or in elderly diabetics. To address this issue, high-dose influenza vaccines (containing four times the antigen of standard doses) and adjuvanted influenza vaccines (e.g., MF59-adjuvanted) have been approved for adults aged 65 years and older [90,95]. Although some open-label, randomized, controlled trials have shown that high-dose inactivated influenza vaccines did not demonstrate a significantly better effect in reducing the incidence of hospitalization due to influenza or pneumonia compared to standard-dose vaccines [90]. More randomized trials have shown that influenza vaccination can significantly reduce the incidence, hospitalization rate, and mortality rate among diabetic patients during influenza epidemic periods [91,92,93,94]. However, there is extremely limited data on the efficacy of high-dose vaccines or adjuvant vaccines specifically targeting diabetes patients rather than those solely of advanced age. Therefore, whether diabetes itself constitutes an independent indication for recommending high-dose or adjuvanted influenza vaccines remains unresolved. A notable area of controversy is whether standard-dose influenza vaccines provide adequate protection in diabetic patients. While observational studies generally support vaccine effectiveness, the immunogenicity data are less consistent, particularly in patients with suboptimal glycemic control. The question of whether high-dose or adjuvanted vaccines should be specifically recommended for diabetic patients, independent of age, remains unresolved. Thus, the decision to use enhanced vaccines in younger diabetic patients must be individualized, weighing potential benefits against cost and supply considerations.

Furthermore, influenza vaccination has been shown to reduce the likelihood of *Streptococcus pneumoniae* infection, highlighting the role of influenza virus prevention and control in mitigating pneumococcal infections. According to the recommendations of the American Diabetes Association, patients with diabetes mellitus should receive the pneumococcal polysaccharide vaccine [96]. For patients aged 65 years or older who have been previously vaccinated, a revaccination is required if more than five years have elapsed since the last dose. Individuals with diabetes between the ages of 2 and 64 years should receive the 23-valent pneumococcal polysaccharide vaccine (PPV23). The study indicates that for elderly diabetic patients aged 75 and above, PPV23 vaccination is effective in preventing pneumococcal diseases and reducing medical resource utilization. Concurrent administration of both influenza and pneumococcal vaccines yields better outcomes compared to receiving PPV23 alone [97]. Currently, the vaccination rate for PPV23 among non-elderly adults with diabetes remains low. Efforts should be intensified to raise awareness, ensuring that more non-elderly diabetic patients understand the significance of PPV23 for their personal health [98].

### 5.2. Treatment Strategy: Comprehensive Multi-Channel Intervention

The treatment principles outlined below integrate evidence from clinical practice guidelines for community-acquired pneumonia and influenza management, with specific considerations for diabetic patients extrapolated from pharmacological and pathophysiological studies. Where evidence specific to diabetic populations is limited, recommendations are proposed as expert consensus-based inferences.

For patients with T2DM complicated by viral and bacterial infections, individualized blood glucose management should be implemented [99]. If the patient was on oral hypoglycemic agents prior to admission with relatively stable blood glucose levels, consideration may be given to continuing the pre-admission hypoglycemic regimen [80]. However, close monitoring of blood glucose fluctuations is essential to allow for timely adjustments to the treatment plan. For patients who were using insulin injections for blood glucose control before admission, subcutaneous insulin administration should be continued [99]. For patients requiring intensive care, continuous low-dose intravenous insulin infusion is recommended [99]. During the monitoring period, insulin dosage may be adjusted at any time based on blood glucose fluctuations. Once the condition stabilizes, a gradual transition to subcutaneous insulin injection should be made according to the specific circumstances.

For patients with diabetes complicated by influenza virus infection, prompt antiviral treatment should be administered. Anti-influenza medications such as oseltamivir phosphate and baloxavir should be initiated as early as possible within 48 h of symptom onset [99,100,101]. Even beyond 48 h, they should still be used for severely ill patients. Early antiviral therapy helps reduce viral load, mitigate immunopathological damage, and thereby indirectly lower the risk of secondary bacterial infections. When selecting anti-infective agents, attention must also be paid to the extent of diabetes-related organ impairment, such as diabetic nephropathy.

For diabetic patients infected with influenza virus who develop secondary bacterial infections, aggressive antimicrobial therapy should be administered. Empirical antibiotic selection must be broad-spectrum, timely, and take into full consideration local epidemiology and resistance patterns. Attention should be paid to potential interactions with hypoglycemic agents, which may lead to reduced drug concentrations or abnormal glucose metabolism, among other effects. In diabetic patients, estimated glomerular filtration rate (eGFR) should be assessed before initiating therapy. Antibiotic doses must be adjusted accordingly for renally cleared drugs. It is also recommended to adjust doses dynamically based on renal function stage. This prevents drug accumulation, which can worsen neurotoxicity or nephrotoxicity. Intensified glucose monitoring is also required during therapy. This is especially critical during early treatment and at the infection control turning point. Insulin requirements may decline rapidly after infection resolution. Timely regimen adjustment is necessary to prevent hypoglycemia.

In the United States, β-lactam antibiotics are the first-line treatment of choice for secondary bacterial pneumonia caused by influenza virus [102,103]. The routinely recommended regimens include a third-generation cephalosporin in combination with a macrolide or doxycycline [103]. If there is a risk of MRSA infection or clinically suspected/confirmed MRSA pneumonia, linezolid or vancomycin should be added [104]. When considering fluoroquinolones for empirical therapy, special attention must be paid to abnormal fluctuations in blood glucose levels [105,106].

### 5.3. Novel Therapeutic Avenues from Mechanistic Insights: An Authors’ Perspective

Beyond glycemic control, vaccination, and antimicrobial therapy, emerging mechanistic insights into immunometabolic dysregulation in diabetic hosts suggest additional strategies that may warrant further investigation. The following methods are considered to have potential, but they still need to undergo rigorous preclinical and clinical validation.

Targeting the NLRP3 inflammasome: Chronic NLRP3 inflammasome activation drives persistent low-grade inflammation that predisposes diabetic patients to dysfunctional immune responses during influenza and secondary bacterial infections. Pharmacological inhibition of NLRP3 or its essential component NEK7 has shown therapeutic potential in animal models of type 2 diabetes, and the repurposing of existing agents with favorable safety profiles, such as rociletinib, warrants exploration in the context of influenza–bacterial super-infection [107].

Immunometabolic modulation via macrophage repolarization: Strategies that promote macrophage repolarization toward a pro-resolution M2 phenotype have shown promise in preclinical diabetic infection models. For instance, the antioxidant epigallocatechin-3-gallate (EGCG), delivered via a single nanoplatform, has been shown to facilitate M1-to-M2 repolarization by scavenging excessive ROS, thereby promoting wound healing in diabetic mice with subcutaneous bacterial infections [108]. While still at an experimental stage, such macrophage-targeted immunomodulatory approaches may offer adjunctive benefits in managing diabetic patients with influenza-associated bacterial complications, particularly where wound healing or tissue repair is compromised.

### 5.4. Knowledge Gaps and Priority Areas for Future Research

Several critical knowledge gaps emerge from this critical synthesis, which collectively define a research agenda for improving outcomes in diabetic patients at risk for influenza-associated bacterial infections.

Vaccination. Whether glycemic control modulates vaccine immunogenicity, and whether diabetes, independent of age, constitutes an indication for high-dose or adjuvanted influenza vaccines, remain unresolved due to the absence of adequately powered diabetes-specific trials.

Diagnostics. Biomarkers capable of identifying diabetic patients at highest risk for progressing from influenza to secondary bacterial pneumonia are lacking, hindering personalized prevention and early intervention strategies.

Therapeutics. The optimal timing, choice, and duration of antibiotic therapy in diabetic patients with influenza-associated bacterial pneumonia have not been defined in diabetes-specific populations. Furthermore, whether antiviral therapy confers equivalent benefits in diabetic versus non-diabetic patients has not been rigorously evaluated.

Pathogenesis. The precise molecular mechanisms linking hyperglycemia to macrophage polarization defects and impaired antibacterial responses in the human lung require further elucidation, ideally through analysis of human tissue samples or advanced organoid models.

Addressing these gaps will require coordinated efforts in basic, translational, and clinical research, with particular emphasis on studies that specifically enroll diabetic populations and stratify by key variables such as glycemic control, diabetes duration, and comorbidities.

## 6. Strengths and Limitations of This Review

This review provides a comprehensive synthesis of the current understanding regarding the complex tripartite interactions among diabetes mellitus, influenza virus infection, and secondary bacterial infections. Its primary strength lies in the integration of mechanistic insights from immunology, metabolism, and microbiology into a coherent pathophysiological framework, which may facilitate both basic research and clinical practice by highlighting the interconnected nature of these pathological processes.

Several limitations should be acknowledged to guide appropriate interpretation of the evidence presented. First, as a narrative review rather than a systematic review or meta-analysis, this article did not employ a predefined, comprehensive search strategy or formal quality assessment of included studies. Consequently, the selection and interpretation of the cited literature may be subject to inherent selection bias, and the synthesis reflects the authors’ critical appraisal rather than a quantitative aggregation of available data. Second, the evidence base for many of the mechanisms discussed derives predominantly from animal models and in vitro experimental systems, which may not fully recapitulate the complex pathophysiological milieu of human diabetic patients. The translational relevance of findings from murine influenza–bacterial co-infection models, in particular, requires cautious interpretation given species-specific differences in immune responses and glucose metabolism. Third, clinical data specifically addressing the diabetic population, particularly from randomized controlled trials evaluating vaccine efficacy, antiviral therapy, or antibacterial regimens, remain remarkably scarce. As a result, several recommendations offered in this review are necessarily based on extrapolation from general population data or on mechanistic plausibility rather than on high-level evidence from diabetes-specific studies. Fourth, the dynamic nature of immune–metabolic interactions during the course of infection is inherently challenging to capture in a static review format, and the temporal sequence and causal relationships among the discussed mechanisms remain areas of ongoing investigation.

Despite these limitations, this review identifies several critical knowledge gaps that collectively define a priority research agenda. We have endeavored to provide a balanced and transparent account of the available evidence, explicitly distinguishing well-established findings from emerging hypotheses and areas of uncertainty. Validating and refining our proposed framework will require future studies in diabetes-specific cohorts, ideally with prospective longitudinal designs and translational endpoints that link mechanistic insights to clinical outcomes, ultimately improving prevention and management in this high-risk population.

## 7. Conclusions

**Figure 5:**
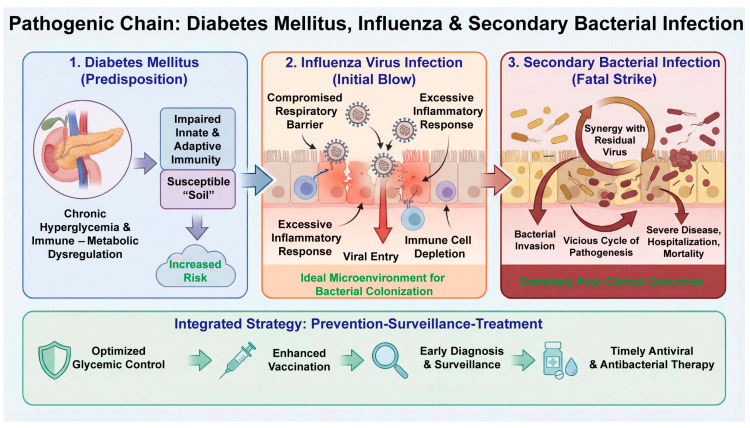
Pathogenic chain: diabetes mellitus, influenza virus infection and secondary bacterial infection. Chronic hyperglycemia and associated immune–metabolic dysregulation impair both innate and adaptive immune responses. Influenza virus infection further compromises the respiratory epithelial barrier, triggers excessive inflammatory responses, and depletes key immune effector cells, thereby creating a permissive microenvironment for bacterial colonization. Subsequent bacterial infection synergizes with viral injury, establishing a vicious cycle of pathogenesis. Clinical management is fraught with challenges, including diagnostic difficulty and therapeutic complexity. Optimized glycemic control, enhanced vaccination coverage, and timely, evidence-based administration of antiviral and antibacterial agents constitute an integrated approach that is fundamental to effective clinical management. The figure was created using PicDoc Web version and Adobe Illustrator 2023 software.

In summary, diabetes mellitus, influenza virus, and secondary bacterial infections form a complex pathogenic triad wherein diabetes establishes a susceptible metabolic and immunological baseline, influenza virus inflicts the primary insult on respiratory defenses, and secondary bacteria exploit this compromised environment to cause severe superinfection (Figure 5). The cumulative pathogenicity of this cascade results in disproportionately poor clinical outcomes in diabetic patients. Effective management requires an integrated ‘prevention–surveillance–treatment’ strategy. Future research should focus on elucidating the precise molecular mechanisms underlying this triad and developing precision medicine approaches tailored to this high-risk population, including dedicated vaccine trials and biomarker-driven therapeutic algorithms.

## Figures and Tables

**Table 1:** Summary of prevention and treatment strategies for influenza and secondary bacterial infections in patients with diabetes mellitus.

Domain	Strategy	Specific Measures/Recommendations	Key Considerations	References
Prevention	Glycemic optimization	Maintain HbA1c targets per individualized guidelines.	Metformin associated with lower influenza hospitalization (retrospective data); SGLT2 inhibitors associated with reduced pneumonia/sepsis risk (real-world evidence) but caution for urogenital infections.	[87,88,89]
	Influenza vaccination	Annual seasonal influenza vaccine (standard-dose).	Standard-dose efficacy may be suboptimal in poorly controlled or elderly diabetic patients.	[90,91,92,93,94]
	Enhanced influenza vaccines	High-dose or MF59-adjuvanted vaccines (approved for ≥65 years).	Whether diabetes independently indicates enhanced vaccines remains unresolved; decision should be individualized.	[90,95]
	Pneumococcal vaccination	PPV23 for all diabetic patients aged 2–64 years; revaccination for ≥65 years if >5 years since last dose.	Simultaneous vaccination with the influenza vaccine and the pneumococcal vaccine can produce synergistic benefits.	[96,97,98]
Treatment	Glycemic management	Continue pre-admission regimen if stable; insulin for hospitalized patients; IV insulin infusion for ICU settings.	Monitor glucose fluctuations closely; adjust insulin promptly at infection resolution to avoid hypoglycemia.	[99]
	Antiviral therapy	Oseltamivir or baloxavir within 48 h of symptom onset (even beyond for severe cases).	Early therapy reduces viral load and may lower secondary bacterial infection risk.	[99,100,101]
	Antibacterial therapy	Empirical broad-spectrum antibiotics (β-lactam + macrolide/doxycycline); add MRSA coverage if suspected/confirmed.	Adjust doses for renal function (eGFR); monitor for hypoglycemic interactions with fluoroquinolones.	[102,103,104,105,106]
Emerging/experimental	NLRP3 inflammasome inhibition	Pharmacological inhibition of NLRP3 or NEK7.	Preclinical only; requires fine-tuning to avoid compromising antimicrobial immunity.	[107]
	Macrophage repolarization	EGCG or other antioxidants to promote M1-to-M2 repolarization.	Experimental (animal models); potential for wound healing and tissue repair.	[108]

## Data Availability

No new data were created or analyzed in this study. Data sharing is not applicable to this article.
